# Photoresist Design for Elastomeric Light Tunable Photonic Devices

**DOI:** 10.3390/ma9070525

**Published:** 2016-06-29

**Authors:** Sara Nocentini, Daniele Martella, Camilla Parmeggiani, Diederik S. Wiersma

**Affiliations:** 1 European Laboratory for Non-Linear Spectroscopy (LENS), University of Florence, via Nello Carrara 1, 50019 Sesto Fiorentino, Italy; nocentini@lens.unifi.it (S.N.); martella@lens.unifi.it (D.M.); wiersma@lens.unifi.it (D.S.W.); 2 Department of Chemistry “Ugo Schiff”, University of Florence, via Della Lastruccia 3-13, 50019 Sesto Fiorentino, Italy; 3 CNR-INO, Sede Secondaria di Sesto Fiorentino, via Nello Carrara 1, 50019 Sesto Fiorentino, Italy

**Keywords:** liquid crystalline elastomers, direct laser writing, azobenzene containing polymers

## Abstract

An increasing interest in tunable photonic structures is growing within the photonic community. The usage of Liquid Crystalline Elastomer (LCE) structures in the micro-scale has been motivated by the potential to remotely control their properties. In order to design elastic photonic structures with a three-dimensional lithographic technique, an analysis of the different mixtures used in the micro-printing process is required. Previously reported LCE microstructures suffer damage and strong swelling as a limiting factor of resolution. In this article, we reported a detailed study on the writing process with four liquid crystalline photoresists, in which the percentage of crosslinker is gradually increased. The experiments reveal that exploiting the crosslinking degree is a possible means in which to obtain suspended lines with good resolution, quite good rigidity, and good elasticity, thereby preserving the possibility of deformation by light irradiation.

## 1. Introduction

The miniaturization of many scientific devices is becoming a more and more attractive possibility. Recent progress, both in the lithographic techniques and in the material science, opens up the potential to model complex structures in the micro-scale and to functionalize the material according to the specific application requirements. In this field, polymer photonics is witnessing an increased interest thanks to the good optical and controllable properties of the polymers. The most relevant advantages of polymers are the possibility of tuning their behavior by chemically controlling the monomer structure and modeling their shape by different lithographic techniques [1]. Soft lithography—and, in particular, the two-photon polymerization technique—allows for the creation of different micro- and nano-scale objects with a desired three dimensional (3D) shape. Different commercially available polymeric formulations can be used [2]. The results of this technique are very appealing not only for producing optical devices [3,4,5] but also in different areas, such as micro-robotics [6,7] or biomedicine and tissue engineering [8].

Regarding the material design for polymer photonics, research efforts have been mainly concentrated on the improvement of the spatial resolution and the obtainment of low-shrinkage structures [9] while, only very recently, the potential to pattern gradient-index materials has been studied [10]. Currently, a great challenge centers on the fabrication of responsive materials that can be employed in the realization of tunable devices. The potential to modulate typical optical features, such as the resonant wavelength for a cavity or the band-gap for photonic crystals, enlarges the area of interest of such structures and allows for the further potential to dynamically act on their optical response.

Within stimuli responsive polymers, our group described how to create 3D liquid crystalline elastomeric (LCE) microstructures that are able to deform from irradiation with light [11]. These materials, that combine the properties of elastomeric polymers with those of liquid crystals, are well known as artificial muscles and allow for reversible deformations in responses to external stimuli as a consequence of the liquid crystal to isotropic phase transition [12,13,14]. The different alignments determine the type of material deformation [15], and we recently demonstrated a two-step fabrication process for the realization of nano-scale patterns sequentially used for controlling the structure local movements depending on the engineered mesogen alignment [16]. At the same time, changing the LCE composition is a possible means to trigger such shape-changes, which can be accomplished through variation of the temperature, irradiation with light [17,18], or the application of electric [19] and magnetic fields [20].

To pattern the LCE in the micro-scale, we employed the Direct Laser Writing (DLW) method, a photolithographic technique based on point by point two-photon polymerization inside a liquid resin droplet (photoresist). Precise control of the laser position in respect to the polymerization cell allows for the creation of desired 3D structures with nano-scale features [10]. To integrate the LCE synthesis with this setup, we need to exploit a photo-activated reaction, such as photopolymerization of acrylate/metacrylate groups [11] or thiol-yne click chemistry [21]. In this paper, polymerization of acrylate-based mesogens was successfully used to create 3D microstructures. Demonstration of this method can be found for micro-robotic structures [7] and tunable optical cavities [22].

The potential applications of the polymer combined with the DLW technique open up the ability to create more complex photonic devices tunable by light. Management of the softness of these elastic materials, in contrast with the rigidity and high resolution often requested, will give access to more complex geometries of photonic devices. This is the reason behind this detailed study on the LCE resolution and properties in the micro-scale. In this article, we investigated the fabrication of LCE microstructures for different liquid crystalline mixture compositions. For each photoresist, the writing conditions are studied and the possibility to create free-standing structures is demonstrated. In the last part, the structure deformation by irradiation with a green laser is shown and the properties of the different mixtures are compared.

## 2. Results and Discussion

### 2.1. Photoresist Design and Preparation

**Figure 1:**
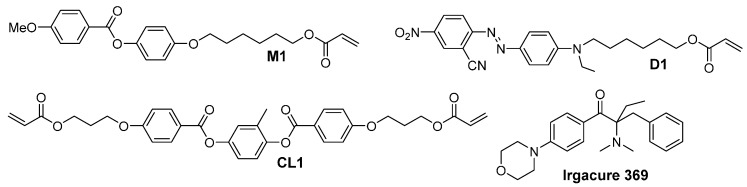
Monomer structures.

Preparation of a photoresist to create LCE structures requires the use of at least three components: a mesogen, responsible of the material alignment; a crosslinker, which allows the formation of a network with an elastic mechanic response; and a photoinitiator, to achieve the spatial control of the radical reaction. The first two components need to be functionalized with one or two photopolymerizable groups, such as the acrylate group. In this study, in order to obtain a photoresponsive material, a dye, such as an azobenzene molecule [18], is added in the monomeric formulations. Molecules employed in this study are depicted in Figure 1.

Photoresists are noted in this article as **PR-a** where *a* is the percentage of the crosslinker. All mixtures contain 1% mol of the initiator Irgacure 369 and 1% mol of the azo-dye **D1** according to our previous experience on DLW with LCEs [11]. Detailed composition of the photoresists is shown in Table 1.

Varying the molar mass ratio of the monomer (**M1**) and cross-linker (**CL1**), we achieve a different crosslinking degree that leads to the control of the structure rigidity. Polarized optical microscopy (POM) shows that the mixtures melt directly in the isotropic phase between 50 and 70 °C, depending on the crosslinker percentage, and they show a very broad range of temperature for the nematic phase on cooling. These mixtures are also liquid crystalline at room temperature and crystallization occurs only after several hours. The integrity of each monomer mixture has been evaluated under the POM for several hours up to 80° verifying that the infiltration process in the liquid crystalline cell can be performed up to this temperature without any undesired polymerizations.

### 2.2. DLW Characterization

The DLW system is constituted by a femtosecond laser directly focused inside a liquid resin droplet (photoresist). Thanks to the non-linear process, the two-photon absorption occurs only around the focal point volume, affording the polymerization of a material voxel. To control the alignment, the liquid crystalline mixtures are infiltrated in homeotropic cells in this study. The laser beam is then focused at the glass/mixture interface and the polymerization starts ensuring that the micro-structures are anchored to the glass surface.

Many parameters are involved in the DLW technique. The repetition rate has been set at 100 MHz, since it has been demonstrated that high repetition rates and short pulses lead to high-resolution structures [23,24]. The mechanism behind the polymerization process, within this repetition rate range, has to be attributed to a multi-photon activation of the photoinitiator [24]. Moreover, for Irgacure 369, it has been demonstrated that a high repetition rate leads to a higher dynamic range [24]. Once this parameter is fixed, writing speed and laser power play the dominant role in the writing conditions. We evaluate the resolution and rigidity properties as a function of these DLW variables using empirical tests for the different mixtures.

In respect to other polymers, the characterization of the voxel dimensions is particularly critical because of the soft nature of the LCEs and the high percentage of swelling of the unpolymerized monomers inside the written structures. After the writing step, the unpolymerized monomers are removed with a development bath in hot 2-propanol. This choice is motivated by the reduced degree of swelling of this organic solvent in respect to toluene, previously used for the development of this polymer [11], and Propylene glycol methyl ether acetate (PGMEA). However, since the monomers are low soluble in 2-propanol, heating above 50 °C is required to completely dissolve the unpolymerized material without any degradation of the polymerized structures.

#### 2.2.1. Polymerization Threshold

**Figure 2:**
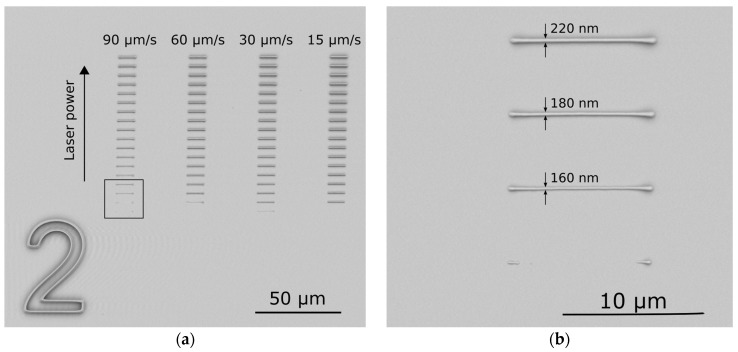
Scanning electron microscope (SEM) image of the writing tests for the threshold power for **PR-20**: laser power and writing speed dependence. (**a**) Laser power is varied from less than 1 mW up to 10 mW, adding 0.44 mW from one segment to the next with each increase in the *y*-position; (**b**) Detail of the threshold lines, zoom of the square in the (**a**) image.

The polymerization threshold is defined as the lowest power able to create a well-defined polymeric line at the glass-resist interface after the development process. This parameter depends on several effects, such as monomer quenching, oxygen quenching, and the different reactivity of each of the radicals and monomers. To experimentally determine these values, writing tests at the glass-resist interface have been performed while varying the writing speed and the laser power. Figure 2a shows the results for mixture **PR-20**. Line patterns are written while increasing the laser power value for different writing speeds ranging from 15 up to 90 µm/s. The indicated power values are measured at the objective position.

From this calibration, we observed that while varying the writing speed that the polymerization threshold remains the same, with a fluctuation of 1%–2% around that laser power value. In Figure 2b the effect of the acceleration and deceleration time of the laser pencil beam can be observed. In fact, to reach the constant writing speed, the piezo translation stage needs a certain acceleration/deceleration time. Consequently, the energy deposited at the beginning and at the end of the segment is higher and leads to a partial polymerization at the ends. Table 2 summarizes the polymerization thresholds: no correlation between these values and the cross-linker percentages can be deduced. Above a certain energy density accumulated in the mixture, polymerization is not well controlled and some micro-explosions, characterized by air bubble formations, can occur. A damage threshold is difficult to define because of imperfections and inhomogeneities within the mixtures. A possible explanation for the explosion occurrence can be found in the presence of one-photon absorption from the impurities leading to locally incurred damage to the polymerized structure [24]. In our case, explosions are more frequent above 16 mW, showing a broad dynamic range.

A first evaluation of the structure resolution can be extrapolated as the minimum size of the voxel minor axis that can be fabricated for each laser power and writing speed. With all the mixtures, the minimum feature size that we were able to obtain at threshold is 160 nm (Figure 2b).

**Figure 3:**
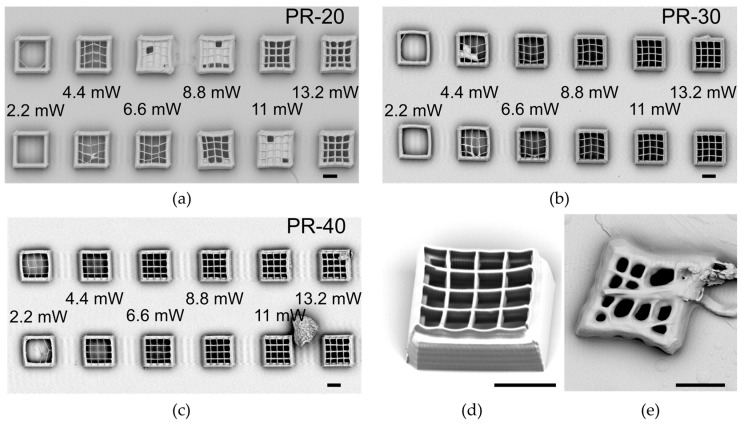
Writing tests for the rigidity and rod dimensions at varying laser powers and writing speeds (90 µm/s for the first row and 60 µm/s for the second row). In the SEM images are shown: (**a**) **PR-20**; (**b**) **PR-30**; (**c**) **PR-40**; (**d**) A grid of **PR-40** realized with a writing speed of 90 µm/s and a laser power of 12.9 mW; (**e**) A grid realized with **PR-10**, a writing speed of 90 µm/s, and a laser power of 12.9 mW. The scale bar is 10 µm.

To increase the structure rigidity, we can act not only on the molar percentage of the crosslinker but also on the writing parameters. Increasing the laser power and decreasing the writing speed, the crosslinking density increases, thereby reducing the possibility of the surrounding monomers swelling inside the polymerized structures [10]. This effect is relevant especially for the fabrication of suspended lines required for 3D photonic crystals, like woodpiles. Figure 3 shows the calibration for suspended grids up to a LCE wall for the different mixtures. **PR-10** results in too soft of a polymer to create 3D structures, and no data are reported because the bad quality of the written structures (Figure 3e).

From Figure 3 it is clear that the structures realized with the **PR-20** mixture are softer, with less sharp edges and higher thickness of the rods. Of course, increasing the amount of the crosslinker in the mixture results in the grids being more rigid and straight, as can be observed for the structure realized with **PR-40** in the SEM image reported in Figure 3d and recorded with the samples placed at 45°.

#### 2.2.2. Voxel Dimensions

**Figure 4:**
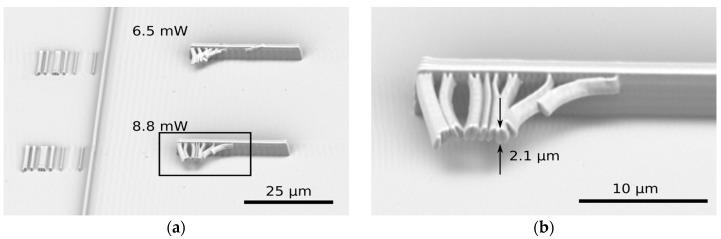
Writing tests for the rod dimensions and resolution evaluation for **PR-40** and a writing speed of 90 µm/s. In (**b**) the zoomed image of the squared detail in image (**a**) can be observed.

To sort out the characteristic voxel dimensions, we analyzed the lines written on the glass-resist interface in order to evaluate the thickness (minor axis of the voxel, as in Figure 2b), and we analyzed those suspended in between two walls or a grid to estimate the height (major axis of the voxel ellipsoid, from Figure 4).

**Figure 5:**
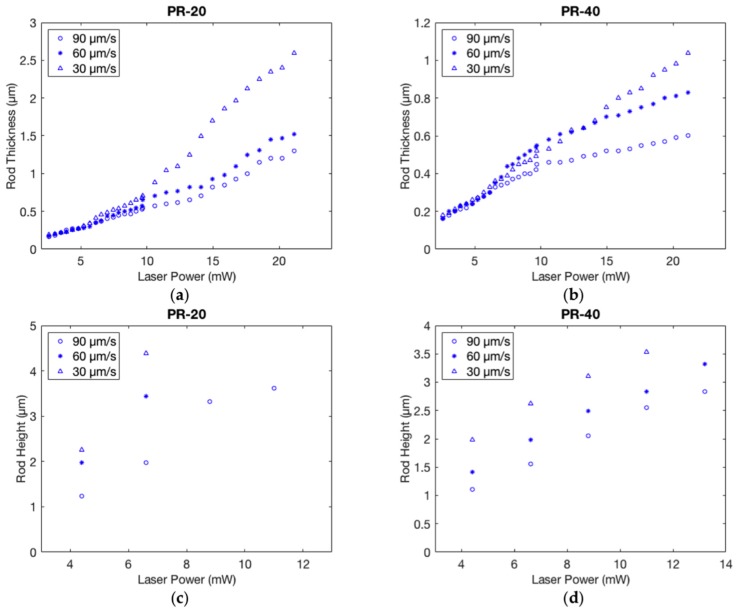
Thicknesses and heights of the voxel depending on the writing speed and the laser power. The values are reported for **PR-20** in (**a**,**c**) and **PR-40** in (**b**,**d**).

Voxel dimensions as a function of the writing laser powers are reported in Figure 5 for **PR-20** and **PR-40** mixtures, and in Figure S1 for the other mixtures.

First of all, it can be observed that there is not a linear correlation between these two physical quantities as also expected for the relationship between the laser irradiation and degree of conversion [10].

The percentage of the crosslinker highly affects the dimension of the polymerized rods above a certain laser power value especially for lower writing speeds. In fact, the thicknesses for **PR-20** (Figure 5a) realized at 30 µm/s speed reach 2.5 µm for a laser power of 20 mW, which is more than double in respect to the value for the **PR-40** (Figure 5b). Moreover, it is particularly significant how the voxel height in **PR-20** increases while decreasing the writing speed. Even if the thickness for the different writing speeds differs by only around 100 nm, the difference in height varies from 2.3 to 4.4 µm. This behavior is consistent with the values reported in the literature where it is reported how an increasing writing power results in voxel lateral size saturation while the voxel height keeps increasing [25]. For less crosslinked mixtures, the higher mobility of the monomers emphasizes this unbalance, leading to thicker and taller rods. Defining the aspect ratio of the ellipsoid as the ratio between the major axis over the minor one, we observed, from Table 3, how for slower writing speeds it reaches a value of 10 for **PR-20** and 7.7 for **PR-40**. For even higher writing speeds, the aspect ratio does not decrease below 5, while for commercial photoresist, like the IP-Dip/Ip-l (Nanoscribe GmbH), the aspect ratio is 2.7 for the writing speed of 100 µm/s [4]. With LCEs, the higher aspect ratio can be attributed to the higher refractive index value with respect to the commercial photoresist previously used (Ip-l). In fact, the dependence of the numerical aperture on the refractive index influences the elongated shape of the voxel resulting in a higher aspect ratio. The probability of two-photon absorption is proportional to the squared value of the electric field intensity that linearly depends on the refractive index value. With a fixed value of the electric field, the two photon absorption probability therefore scales with the squared value of the refractive index influencing the voxel dimension. Comparing this resolution value with those previously reported (300 nm) [11], for homogenous alignment the resolution determination is even more complex to analyze due to the ordinary and extraordinary refractive index contributions.

Fixing the writing velocity, we consider the variation of the aspect ratio as a function of the employed laser power. From Table 4, we can observe that the aspect ratio is bigger for **PR-20** in respect to **PR-40**, but that the values remain almost comparable. Writing speed is then the parameter that exerts a greater influence on the aspect ratio in LCE microstructures.

### 2.3. Light Actuation of the Microstructures

The ability to control the microstructure deformations by irradiation with light is the feature that could make this material unique for further developments in photonics. The phase transition mechanism for azobenzene containing polymer is different depending on the aromatic ring substituents, and this mainly affects the lifetime of the cis azobenzene and its percentage in the photostationary state [26]. In particular, the compound chosen for this study, **D1**, acts mainly as a nanoscale heater which is able to induce the nematic to isotropic phase transition after absorption of light in the visible region. However, even if part of this energy could also be dissipated for the *trans* to *cis* isomerization, the thermal effect is largely predominant with **D1** at low percentages in the mixtures [11]. Photothermal actuation results in greater appeal for photonics due to the LCE characteristic response time, in the millisecond scale [22], which allows for the true real time tunability of devices.

The fabricated microstructures present a nematic alignment associated with a typical contraction along the alignment direction and the expansion in the perpendicular plane. Such deformation is consistent with a prolate conformation of the polymer, in which chains have a preferential alignment parallel to the mesogenic cores, as reported for other polymers that have shown only nematic behavior [13,27].

**Figure 6:**
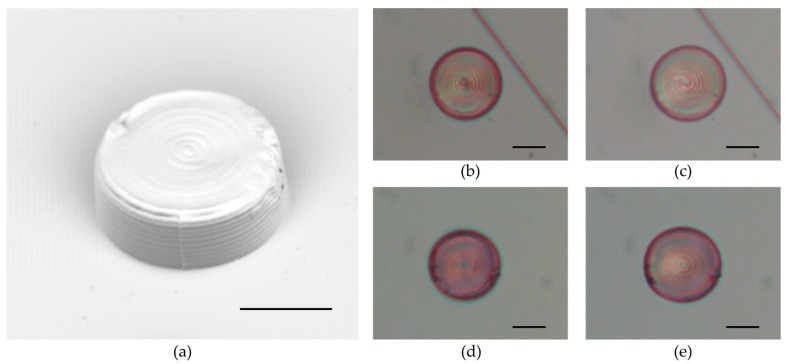
Light induced deformation. (**a**) SEM image of a cylinder made by **PR-40**; (**b**,**d**) optical image of a cylinder made respectively by **PR-20** and **PR-40** before irradiation; (**c**,**e**) optical image of a cylinder made respectively by **PR-20** and **PR-40** during irradiation with green light. Scale bar: 15 μm.

The light response of our photoresists has been tested through cylindrical structures (13 μm height, 30 μm diameter) fabricated in homeotropic cells (writing speed 90 µm/s, laser power 8.5 mW). The cylinders were irradiated with a green laser focused on the bottom with a 20× objective, and the light intensity was varied using a neutral density wheel filter. The focal spot covered the whole cylinder surface. The deformations were observed from the top. Since the structures were attached to the glasses, even if the contraction occurred inside the entire volume [15], the observed deformation created a reversed truncated cone shape rather than a shrunk cylinder due to the strain forces of the substrate. Examples of light deformation are shown in Figure 6, while the deformation of all samples is shown in the Video S1.

Different actuation threshold powers are observed: in **PR-10**, deformation starts from irradiation with 2.13 mW and keeps increasing up to 11.6 mW. Above this laser power, the cylinder is burned by the laser. The employed minimum power, for a laser spot of 15 µm diameter, corresponds to a power flux of 3 W/mm^2^. The maximum expansion reached by the top of the structure was around 10% of the initial area. **PR-20** and **PR-30** exhibit a similar behavior with an actuation threshold at around 4.19 mW and maximum expansion at 11.6 mW. For **PR-40** the actuation threshold was a bit higher (5.89 mW) but the maximum deformation was reached at the same power. The extent of the areal expansion on the top of the cylinder at 11.6 mW was comparable for the different samples and it was around 10%. We could not exclude the possibility that a higher crosslinking degree affects the extent of the contraction, but a correct estimation of this effect required the evaluation of freestanding microstructures. The presented data outline an increase of the power threshold for the actuation by increasing the crosslinker percentage even if all samples show comparable maximum deformation.

## 3. Materials and Methods

### 3.1. Materials

**M1** and **CL1** were purchased from Synthon Chemical (SYNTHON Chemicals GmbH & Co. KG, Wolfen, Germany), Irgacure 369 was purchased from Sigma Aldrich (Sigma Aldrich SRL, Milano, Italy), and **D1** was prepared as previously described [11].

### 3.2. Cell Preparation

Cells were prepared by means of polyimide (PI1211 Nissan Chemical Industries, Tokyo, Japan) coated glasses with a 20 μm spacer. The coating was chosen in order to reach the homeotropic alignment of the liquid crystalline photoresist. The mixtures were melted on a hot plate at 70 °C and then, infiltrated for capillarity in the cells. Afterwards, the cells were cooled down to room temperature, and the liquid crystalline alignment was checked with an inverted microscope (Zeiss, Axio Observer A1, Jena, Germany) with cross polarizers. The cell was glued into a sample holder for the inverted microscope of the DLW system.

### 3.3. Direct Laser Writing Setup

Two-photon absorption polymerization was induced by a focused laser beam from a 780 nm femtosecond laser in a commercial DLW workstation (Photonic Professional, Nanoscribe GmbH, Eggenstein-Leopoldshafen, Germany). We used a pulsed erbium doped femtosecond (120 fs) fiber laser source at a center wavelength of 780 nm with a repetition rate of 100 MHz integrated in a commercial system Nanoscribe GmbH (Nanoscribe Photonic Professional). The laser beam was tightly focused through an immersion oil objective (Plan Apocromat, NA 1.3, Carl Zeiss, Oberkochen, Germany) into a cell.

### 3.4. Structure Development

After the writing process, the LCE cell was removed from the sample holder, opened with a blade, and put in a bath of 2-propanol at 60 °C. After 20 min, the glass was dried with clean air and we verified the complete removal of the unpolymerized monomers through an optical microscope. The bath was repeated if some monomers were still present around the structures.

### 3.5. Structure Characterization

A scanning electron microscope (PHENOM-World, Eindhoven, The Netherlands) was used to observe the LCE structures after sputter-coating them with a 10 nm gold layer. Light induced deformations were observed when the structures were illuminated with a DPSS 532 nm laser through a 20×, 0.4 NA (Plan Achromat, Olympus, Tokyo, Japan) objective placed above the sample structure and the movement movies were recorded by a CMOS camera (frame rate 25.8 fps).

## 4. Conclusions

Photosensitive elastic structures emerged as good optically tunable photonic components. In this article, we proposed a LCE composition exploration in the microscale using the lithographic technique of DLW. Depending on the desired photonic structure, the best writing condition can be identified on the basis of this study, keeping into account that resolution, rigidity, and light deformation have to be balanced in order to optimize the final device. For each mixture, the polymerization degree should be increased as a function of the laser power and writing speed, thereby leading to lower shrinkage of the structures during the fabrication process. Unfortunately, high exposure energy produces high voxel dimensions and the photoresist birefringence also has to be taken into account to determine the voxel aspect ratio. Increasing the crosslinker percentage is a good strategy for obtaining freestanding polymeric objects with nanometric feature sizes, and, in particular, photoresist **PR-40** is the more promising option for creating suspended elements. In contrast, the resolution at threshold is almost invariant for the different writing speeds and mixtures. The contractive properties are retained for all mixtures even if the crosslinker percentage affects the actuation threshold power. In conclusion, we underline that the proposed LCE mixtures are good matrixes for the realization of tunable photo-elastic photonic devices with the Direct Laser Writing technique, thanks to their high resolution, low degree of swelling, and broad range of parameters.

## Figures and Tables

**Table 1:** Photoresists composition (mol % of the total mixture).

Photoresist	M1	CL1	D1	Irgacure 369
**PR-10**	88	10	1	1
**PR-20**	78	20	1	1
**PR-30**	68	30	1	1
**PR-40**	58	40	1	1

**Table 2:** Photoresist polymerization thresholds.

Photoresist	Polymerization Threshold
**PR-10**	3.52 mW
**PR-20**	2.2 mW
**PR-30**	3.52 mW
**PR-40**	2.64 mW

**Table 3:** Voxel aspect ratio for a laser power of 6.6 mW.

Writing Speed	PR-20	PR-40
30 µm/s	10.5	7.7
60 µm/s	9.8	7.1
90 µm/s	5.35	5

**Table 4:** Voxel aspect ratio for a writing speed of 90 µm/s.

Laser Power	PR-20	PR-40
4.4 mW	4.92	5.2
6.6 mW	5.35	4.9
8.8 mW	7	5.4
11 mW	6.6	6.2
13.2 mW	-	6.2

## Supplementary Materials

The following are available online at www.mdpi.com/1996-1944/9/7/525/s1. Figure S1: Thicknesses and heights of the voxel for **PR-10** and **PR-30**; Video S1: Voxel dimensions evaluation and cylinder deformation evidence.

## Abbreviations

The following abbreviations are used in this manuscript:

DLW	Direct laser writing
LCE	Liquid crystalline elastomer
POM	Polarized optical microscopy
PGMEA	Propylene glycol methyl ether acetate
SEM	Scanning electron microscope
NA	Numerical aperture

## References

[1] Hohmann J.K., Renner M., Waller E.H., von Freymann G. (2015). Three-dimensional μ-printing: An enabling technology. Adv. Opt. Mater..

[2] Maruo S., Fourkas J.T. (2008). Recent progress in multiphoton microfabrication. Laser Photon. Rev..

[3] Schumann M., Bückmann T., Gruhler N., Wegener M., Pernice W. (2014). Hybrid 2D–3D optical devices for integrated optics by direct laser writing. Light Sci. Appl..

[4] Deubel M., von Freymann G., Wegener M., Pereira S., Busch K., Soukoulis C.M. (2004). Direct laser writing of three-dimensional photonic-crystal templates for telecommunications. Nat. Mater..

[5] Grossmann T., Schleede S., Hauser M., Beck T., Thiel M., von Freymann G., Mappes T., Kalt H. (2011). Direct laser writing for active and passive high-Q polymer microdisks on silicon. Opt. Express.

[6] Huang T.-Y., Sakar M.S., Mao A., Petruska A.J., Qiu F., Chen X.-B., Kennedy S., Mooney D., Nelson B.J. (2015). 3D printed microtransporters: Compound micromachines for spatiotemporally controlled delivery of therapeutic agents. Adv. Mater..

[7] Zeng H., Wasylczyk P., Parmeggiani C., Martella D., Burresi M., Wiersma D.S. (2015). Light-fueled microscopic walkers. Adv. Mater..

[8] Nahmias Y., Schwartz R.E., Verfaillie C.M., Odde D.J. (2005). Laser-guided direct writing for three-dimensional tissue engineering. Biotechnol. Bioeng..

[9] Ovsianikov A., Viertl J., Chichkov B., Oubaha M., MacCraith B., Sakellari I., Giakoumaki A., Gray D., Vamvakaki M., Farsari M. (2008). Ultra-low shrinkage hybrid photosensitive material for two-photon polymerization microfabrication. ACS Nano.

[10] Žukauskas A., Matulaitien I., Paipulas D., Niaura G., Malinauskas M., Gadonas R. (2015). Tuning the refractive index in 3D direct laser writing lithography: Towards GRIN microoptics. Laser Photon. Rev..

[11] Zeng H., Martella D., Wasylczyk P., Cerretti G., Lavocat J.C.G., Ho C.H., Parmeggiani C., Wiersma D.S. (2014). High-resolution 3D direct laser writing for liquid-crystalline elastomer microstructures. Adv. Mater..

[12] Finkelmann H., Kock H.-J., Rehage G. (1981). Investigations on liquid crystalline polysiloxanes 3. Liquid crystalline elastomers—A new type of liquid crystalline material. Makromol. Chem. Rapid Commun..

[13] Ohm C., Brehmer M., Zentel R. (2010). Liquid crystalline elastomers as actuators and sensors. Adv. Mater..

[14] Brömmel F., Kramer D., Finkelmann H. (2012). Preparation of liquid crystalline elastomers. Adv. Polym. Sci..

[15] De Haan L.T., Schenning A.P., Broer D.J. (2014). Programmed morphing of liquid crystal networks. Polymer.

[16] Zeng H., Wasylczyk P., Cerretti G., Martella D., Parmeggiani C., Wiersma D.S. (2015). Alignment engineering in liquid crystalline elastomers: Free-form microstructures with multiple functionalities. Appl. Phys. Lett..

[17] Li M.-H., Keller P., Li B., Wang X., Brunet M. (2003). Light-driven side-on nematic elastomer actuators. Adv. Mater..

[18] Ikeda T., Mamiya J.-I., Yu Y. (2007). Photomechanics of liquid-crystalline elastomers and other polymers. Angew. Chem. Int. Ed..

[19] Chambers M., Finkelmann H., Remškar M., Sánchez-Ferrer A., Zalar B., Žumer S. (2009). Liquid crystal elastomer-nanoparticle systems for actuation. J. Mater. Chem..

[20] Winkler M., Kaiser A., Krause S., Finkelmann H., Schmidt A.M. (2010). Liquid crystal elastomers with magnetic actuation. Macromol. Symp..

[21] Martella D., Parmeggiani C., Wiersma D.S., Piñol M., Oriol L. (2015). The first thiol-yne click chemistry approach for the preparation of liquid crystalline elastomers. J. Mater. Chem. C.

[22] Flatae A.M., Burresi M., Zeng H., Nocentini S., Wiegele S., Parmeggiani C., Kalt H., Wiersma D.S. (2015). Optically controlled elastic microcavities. Light Sci. Appl..

[23] Malinauskas M., Danilevičius P., Juodkazis S. (2011). Three-dimensional micro-/nano-structuring via direct write polymerization with picosecond laser pulses. Opt. Express.

[24] Fischer J., Mueller J.B., Kaschke J., Wolf T.J.A., Unterreiner A.-N., Wegener M. (2013). Three-dimensional multi-photon direct laser writing with variable repetition rate. Opt. Express.

[25] Sun H.-B., Tomokazu T., Satoshi K. (2002). Three-dimensional focal spots related to two-photon excitation. Appl. Phys. Lett..

[26] Bandara H.M.D., Burdette S.C. (2012). Photoisomerization in different classes of azobenzene. Chem. Soc. Rev..

[27] Noirez L., Keller P., Cotton J.P. (1995). On the structure and chain conformation of side-chain liquid crystal polymers. Liq. Cryst..

