# Supplementary material for: Photoresist Design for Elastomeric Light Tunable Photonic Devices
Source: Materials (Basel). 2016 Jun 29;9(7):525. doi: 10.3390/ma9070525 (PMC5456896; doi:10.3390/ma9070525)
Supplement: Supplementary file 1 [file materials-09-00525-s001.zip › materials-132861-supplementary-final.pdf]

Supplementary Materials: Photoresist Design for Elastomeric Light Tunable Photonic Devices

Sara Nocentini, Daniele Martella, Camilla Parmeggiani and Diederik S. Wiersma

DLW Characterization: Voxel Dimensions

In order to have a complete characterization for all the mixtures that have been presented in the article, here further data are reported for the voxel dimension for mixture **PR-10** and **PR-30**.

For the first one, there are not any data about the height of the voxel since, due to the softness of the material, it is not possible to realize suspended lines from which evaluate the major axis of the ellipsoidal rods. For **PR-30**, the measurements are reported for the writing speed of 90 µm/s.

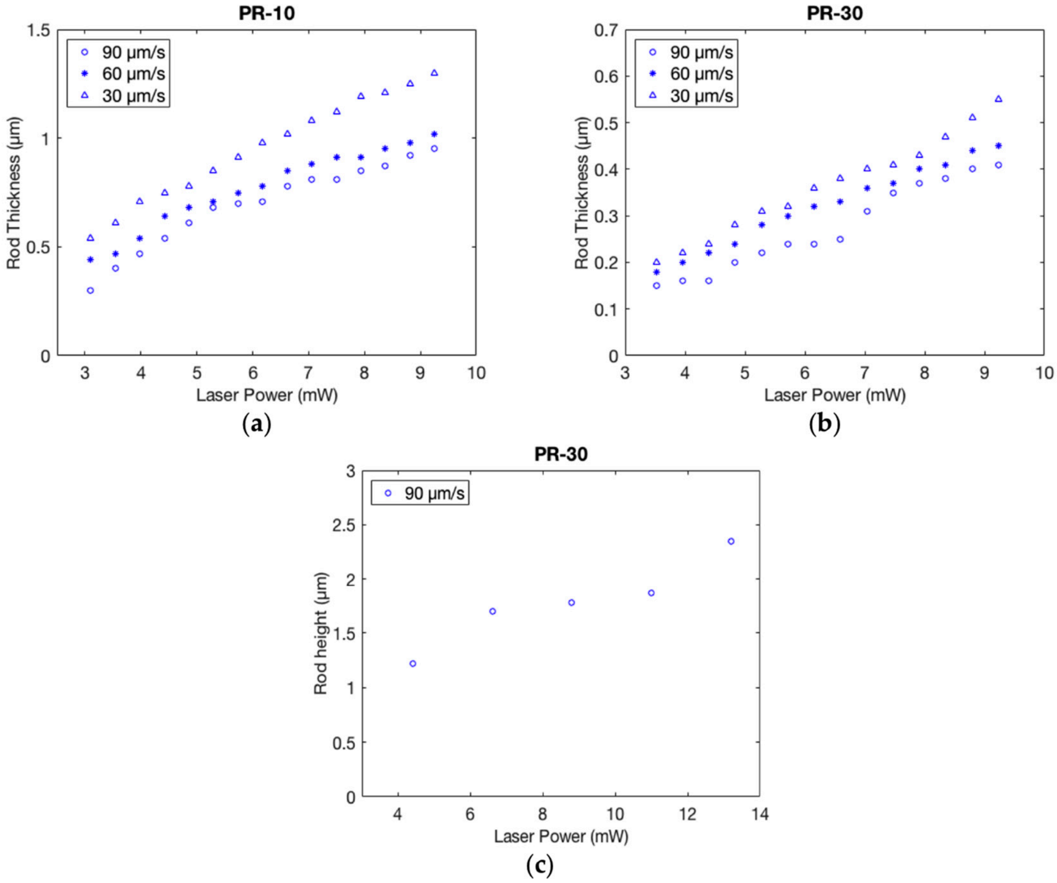

Figure S1. Thicknesses and heights of the voxel depending on the writing speed and the laser power. The values are reported for **PR-10** in (**a**) and **PR-30** in (**b**,**c**).

Light Actuation of the Microstructures

In the Video S1, the cylinder deformation is recorded. The cylinder with a 15 µm radius and a 13 µm height have been realized with the DLW technique with a laser power of 8.5 mW and a writing speed of 90 µm/s with the different mixtures. A green laser focused with a 20× objective has been employed as light stimulus to induce the opto-thermal phase transition. The power has been varied gradually in steps starting from the power value for which the smallest deformation can be appreciated, up to 11.6 mW when the top part of the cylinder reaches the maximum expansion for all the mixtures in analysis. Above this value, for longer exposition, the structures burn.
